# Targeting protein-protein interactions using methods of cheminformatics

**DOI:** 10.1186/1758-2946-4-S1-P3

**Published:** 2012-05-01

**Authors:** Art Cherkasov

**Affiliations:** 1 Department of Urological Sciences, University of British Columbia, Vancouver, BC, V6H 3Z6, Canada

## 

We have recently mapped the protein interaction networks of methicillin-resistant Staphylococcus aureus that revealed its scale-free organization with characteristic presence of highly-connected hub proteins that are critical for bacterial survival [1]. Here we report the discovery of highly selective nanomolar inhibitors for one such hub target - staphylococcal pyruvate kinase. The lead compound has been identified through synergetic combination of methods of high-throughput screening and cheminformatics; its further synthetic modifications resulted in much improved antimicrobial properties. Further lead optimization yielded drug candidates with picomolar activity against methicillin-resistant Staphylococcus aureus.

Considering a notable lack of recent reports on novel antibacterial targets and cognate antibacterial compounds, this study provides a valuable perspective on the development of a new generation of antimicrobials. Equally noteworthy, the results of the current work highlight the importance of cheminformatics-driven exploration of chemical space around initial high throughput screening hits.
